# Enhancing colorectal cancer diagnosis with the ferroptosis marker GPX4 and serum biomarkers: A retrospective analysis and machine learning approach

**DOI:** 10.1016/j.bbrep.2026.102517

**Published:** 2026-02-24

**Authors:** Chao Mei, Jianfeng Shi, Hanxin Liu, Xiao Liu, Xiang Feng, Chenbo Chen, Zixin Wang, Wenjie Pan, Shuo Bai, Li Zhang, Yang Li

**Affiliations:** a The First Affiliated Hospital, Jinzhou Medical University, Jinzhou, Liaoning, 121000, China; b Department of Laboratory Medicine, Fourth Clinical College, China Medical University, Shenyang, Liaoning, 110000, China; c Harbin Medical University, Harbin, Heilongjiang, 150081, China; d Bengbu Medical University, Bengbu, Anhui, 233000, China; e Department of Laboratory Medicine, The Third Affiliated Hospital, Jinzhou Medical University, Jinzhou, Liaoning, 121000, China

**Keywords:** Colorectal cancer, Ferroptosis, GPX4, Machine learning, Immune infiltration

## Abstract

**Background:**

Colorectal cancer (CRC) remains a significant global health burden characterized by high incidence and mortality. Although early diagnosis is crucial for improving prognosis, current screening methods are limited by insufficient sensitivity. Ferroptosis, an iron-dependent form of regulated cell death, is implicated in tumor progression, with Glutathione peroxidase 4 (GPX4) serving as a key regulator in this process.

**Objective:**

This study aims to evaluate the potential of GPX4 as an early diagnostic biomarker for CRC and to construct an integrated predictive model combining ferroptosis-related indicators with machine learning algorithms to enhance diagnostic precision.

**Methods:**

Clinical data and GPX4 gene expression profiles were retrieved from the TCGA-COAD database for survival, differential expression, single-cell transcriptomic, and immune infiltration analyses. Subsequently, GPX4 expression was validated in CRC tissues, adjacent normal tissues, and cell lines with varying metastatic potentials using immunohistochemistry (IHC), qRT-PCR, and Western blotting. Serum levels of carcinoembryonic antigen (CEA), carbohydrate antigen 19-9 (CA19-9), GPX4, and serum iron were quantified in 120 CRC patients and 120 healthy controls. The diagnostic efficacy of individual markers was assessed using receiver operating characteristic (ROC) curves and Kappa analysis. Finally, nine machine learning algorithms were employed to develop a combinatorial diagnostic model based on serum ferroptosis-related indicators, with performance evaluated via ROC and decision curve analysis (DCA).

**Conclusion:**

Ferroptosis plays a critical role in CRC pathogenesis and progression. GPX4 was identified as a robust biomarker for early diagnosis. The integrated machine learning model, incorporating GPX4, CEA, CA19-9, and serum iron, demonstrated superior diagnostic performance compared to conventional markers, offering a promising strategy for the early detection of CRC.

## Highlights

This study has made significant contributions to exploring diagnostic methods for colorectal cancer, highlighting the following key points:

• Critical Role of GPX4 in Colorectal Cancer Survival Status and Immune Cell Infiltration:
  ● It was found that low GPX4 expression was associated with a higher survival rate in colorectal cancer patients, providing new biomarker clues for diagnosis and treatment.
  ● The correlation between GPX4 and various immune cells was demonstrated, and immunohistochemical analysis of cancer tissues and surrounding tissues revealed the relationship between GPX4, tumor immune microenvironment, and tumor parenchyma.
• Innovations in Colorectal Cancer Diagnosis by Combining GPX4 Serum Markers:
  ● The combination of GPX4 with traditional serum markers such as CEA and CA199 significantly improved the diagnostic accuracy of colorectal cancer, offering new insights for clinical diagnosis.
  ● An analysis of combined detection of ferroptosis markers and traditional serum markers presented a novel and more accurate method for colorectal cancer diagnosis.
• Cutting-Edge Application of Machine Learning in Colorectal Cancer Diagnosis:
  ● Utilizing various machine learning algorithms, diagnostic models for colorectal cancer were constructed, achieving high AUC values and demonstrating good predictive performance.
  ● Through machine learning methods, new approaches and possibilities for personalized diagnosis of colorectal cancer were provided, showcasing the potential of technology in the medical field.

These innovative findings not only expand the research scope of colorectal cancer diagnosis but also offer new directions and strategies for early diagnosis and treatment in future clinical practice.

## 1 Introduction

Colorectal cancer (CRC) is a malignant gastrointestinal neoplasm characterized by high global incidence and mortality rates, particularly in developed nations and rapidly developing regions such as China, where its prevalence continues to rise annually [1]. Despite advancements in medical technology, the early detection of CRC remains challenging. The disease is often associated with prolonged treatment cycles and a high propensity for recurrence [2].

Accumulating evidence suggests that tumorigenesis and progression are closely linked to ferroptosis, a novel form of regulated cell death driven by iron-dependent lipid peroxidation. This process is distinct from traditional cell death pathways such as apoptosis, necrosis, and autophagy [3].In recent years, ferroptosis has garnered significant attention in oncology due to its unique mechanism and therapeutic potential [4]. Glutathione peroxidase 4 (GPX4) functions as a central negative regulator of ferroptosis by reducing toxic lipid peroxides. Consequently, cancer cells often exhibit upregulated GPX4 expression to evade ferroptosis, thereby promoting tumor survival and progression. Furthermore, ferrous ions play an essential role in this process, and their dysregulation has been implicated in tumor initiation [[5], [6], [7]].

Machine learning (ML), a fundamental branch of artificial intelligence, focuses on using algorithms to process input data, construct predictive models, and apply them to novel scenarios [8]. Currently, ML algorithms are extensively employed in clinical diagnosis and prognosis. In biomedical research, their applications span diverse fields such as medical imaging [[9], [10], [11], [12], [13], [14]]、pathomics [[15], [16], [17], [18]]、and genomics [[19], [20], [21]], contributing to the development of more accurate and efficient diagnostic tools. Furthermore, diverse omics data can be integrated within ML frameworks, thereby facilitating the clinical translation of multi-omics strategies [[22], [23], [24], [25]]. Consequently, ML plays an indispensable role in advancing clinical research and precision medicine.

Although CRC screening is widely implemented, the sensitivity of established methods—such as conventional imaging and Carcinoembryonic Antigen (CEA) testing—remains suboptimal [26]. Therefore, the present study aimed to systematically evaluate the diagnostic potential of GPX4 by quantifying its expression levels in CRC tissues and serum. Subsequently, we constructed and validated a combined diagnostic model integrating GPX4, serum iron, Carcinoembryonic Antigen (CEA), and Carbohydrate Antigen 19-9 (CA19-9), employing nine machine learning algorithms. This strategy is intended to enhance the sensitivity and accuracy of early CRC screening, establishing a novel biomarker panel for clinical application.

## 2 Methods

### 2.1 Patient

A total of 120 patients diagnosed with CRC at the First Affiliated Hospital of Jinzhou Medical University between October 2021 and April 2023 were enrolled in the CRC group. The inclusion criteria required a confirmed diagnosis via colonoscopic biopsy according to the Chinese Guidelines for the Diagnosis and Comprehensive Treatment of Colorectal Cancer Liver Metastases (2018 Edition); absence of prior surgical resection, radiotherapy, chemotherapy, or targeted therapy; and normal baseline biochemical indices. Patients were excluded if they presented with inflammatory bowel disease, familial adenomatous polyposis or a family history of CRC, severe dysfunction of major organs (liver, heart, or lungs), concurrent malignancies, recent administration of blood products interfering with laboratory results, or severe psychiatric disorders precluding informed consent.

**Fig. 1:**
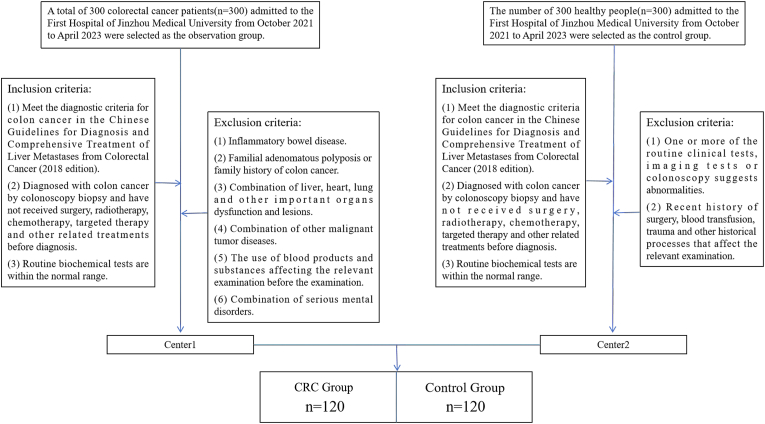
Inclusion and exclusion criteria for the CRC and control groups.

Simultaneously, 120 healthy individuals with normal physical examination results were recruited from the Health Examination Center of the same institution as the Control group. Inclusion criteria for controls included complete clinical records with no pathological findings on imaging or colonoscopy, and no history of hematological disorders, malignancies, or autoimmune diseases. Individuals were excluded if routine screening revealed abnormalities or if they had a recent history of surgery, blood transfusion, or trauma. The detailed enrollment process is illustrated in Fig. 1.

Demographic analysis showed no statistically significant difference in gender distribution between the two groups, while a significant difference in age was observed. The study protocol was approved by the Medical Ethics Committee of Jinzhou Medical University, and informed consent was obtained from all participants.

For the validation of tissue expression, a separate cohort of newly diagnosed CRC patients (2022–2023) was selected. These patients had not received preoperative radiotherapy, chemotherapy, or surgical intervention. Immunohistochemistry, qRT-PCR, and Western blotting were performed to evaluate GPX4 expression in tumor tissues, matched adjacent normal tissues, and CRC cell lines.

### 2.2 Bioinformatic analysis of *GPX4* in CRC

Clinical data, *GPX4* gene expression profiles, and immune infiltration scores for 12 immune cell types in CRC patients were retrieved from The Cancer Genome Atlas Colon Adenocarcinoma (TCGA-COAD) database. Survival, single-cell expression, differential expression, and immune infiltration analyses were conducted using R software (version 4.5.1).

### 2.3 Western blotting

Total protein was extracted from tumor tissues and paired adjacent normal tissues using RIPA lysis buffer (Merck Millipore, 92590). Lysates were resolved by SDS-PAGE and transferred to PVDF membranes (Merck Millipore, ISEQ00010). Membranes were blocked with 10% skimmed milk for 1 h at 37 °C with gentle agitation, followed by overnight incubation with anti-GPX4 primary antibody (1:1000) at 4 °C. After washing with PBS, membranes were incubated with HRP-conjugated secondary antibodies for 1.5 h at room temperature. Protein bands were visualized using an ECL kit (Beyotime, P0018) and imaged via the Bio-Rad ChemiDoc™ MP System. Band intensity was quantified using ImageJ software.

### 2.4 Quantitative real-time PCR (qRT-PCR)

Total RNA was extracted from paired adjacent normal and tumor tissues using an RNA extraction kit (Accurate Biology, AG21017). Reverse transcription was performed using the Evo M-MLV RT Premix for qPCR (Accurate Biology, AG11706). The resulting cDNA was amplified using SYBR Premix Ex Taq™ (Accurate Biology, AG11702) on a Rotor-Gene Q real-time PCR system (Qiagen, Germany). Relative gene expression levels were calculated using the 2^−ΔΔCt^ method [Livak & Schmittgen, 2001]. All reactions were performed in triplicate. The primer sequences used in this study are listed in Table 1, which details gene accession numbers, nucleotide positions, and intron/exon binding sites.

**Table 1:** Primer sequences and amplification details for *GPX4*.

Gene	NCBI Gene Accession Number	Forward Primer (5' → 3′)	Reverse Primer (5' → 3′)	Product Length (bp)	Binding Site
GPX4	NM_001039847.3	TCACCAAGTTTGGACACCGT	ATAGTGGGGCAGGTCCTTCT	117	Exon3/Exon 4

### 2.5 Measurement of blood parameters

Peripheral blood samples were collected and immediately stored at −80 °C. Prior to analysis, samples were thawed at room temperature and centrifuged at 2000–3000 rpm for 20 min to isolate serum. Levels of CEA, CA19-9, serum iron, and GPX4 were quantified. CEA and CA19-9 were measured using chemiluminescence immunoassays, while GPX4 was detected via enzyme-linked immunosorbent assay (ELISA). Serum iron was assessed using standard colorimetric assays. All procedures followed the manufacturers' protocols.Quality control (QC) included blank controls, standard curves R^2^ > 0.99), and duplicate measurements. Samples exceeding the linear range were diluted and re-analyzed. All internal QC results were within established limits.

#### 2.5.1 Quantification of serum GPX4

GPX4 concentration was quantified using a commercial ELISA kit (Cat# JM-0644H1, Lot# 20240719; Ifang Biological). A standard curve (1.25–20 pmol/mL) was generated by serial dilution. Serum samples were diluted 1:5. Aliquots (50 μL) were incubated in pre-coated wells at 37 °C for 30 min. Plates were washed five times with 1 × wash buffer, followed by a 10-min incubation with substrate solution (Chromogen A/B mixture) at 37 °C in the dark. The reaction was terminated with stop solution, and absorbance was measured at 450 nm. Concentrations were calculated using a four-parameter logistic (4-PL) calibration curve.

#### 2.5.2 Quantification of tumor markers

CEA and CA19-9 levels were measured on an Abbott Architect i2000 chemiluminescence immunoassay analyzer. Reagents included the CEA kit (Cat# 7K68, Lot# 76347FZ00; Calibrator 7K68-02) and CA19-9 kit (Cat# 2K91, Lot# 76655FP00; Calibrator 2K91-02). Quality control (QC) was performed using the Architect Immunoassay Multikit Control and Bio-Rad Lyphochek Tumor Marker Plus Control (Cat# 368X). The laboratory participated in the external quality assessment (EQA) schemes of the National Center for Clinical Laboratories (NCCL).

#### 2.5.3 Quantification of serum iron

Serum iron was quantified on an Abbott Architect c16000 biochemical analyzer using the Ferene method. The assay utilized the specific iron reagent kit (Cat# 07K63-20) and multi-parameter calibrators (Cat# 07K60-01). Daily QC was conducted using multi-parameter biochemical controls to ensure assay reliability.

### 2.6 Statistical analysis

Statistical analyses were performed using SPSS (version 27.0) and R (version 4.5.1). The normality of continuous variables was assessed via the Shapiro–Wilk test. Normally distributed data are presented as mean ± standard deviation (SD), while non-normally distributed data are expressed as median [interquartile range (IQR)]. Group comparisons were conducted using the independent samples *t*-test or Mann–Whitney *U* test, depending on data distribution. Categorical variables were analyzed using the Chi-square (X^2^) test or Fisher's exact test.

Diagnostic efficacy was evaluated by generating Receiver Operating Characteristic (ROC) curves, from which the Area Under the Curve (AUC), optimal cutoff values, sensitivity, and specificity were derived. Independent diagnostic factors were identified using binary logistic regression. The Kappa test was employed to assess diagnostic agreement.

To complement the conventional metrics, a symmetric discriminative index, P_sym_ = max (AUC, 1 − AUC), was calculated to provide an intuitive measure of discriminative ability. This index served solely as a supplementary reference; final model performance was primarily evaluated based on the AUC, sensitivity, and specificity. Statistical significance was defined as two-tailed P < 0.05.

### 2.7 Machine learning

In this study, both preliminary and formal experimental procedures were implemented. As the experimental steps were consistent across phases aside from differences in sample sizes, the following description focuses on the formal experimental process.

#### 2.7.1 Machine learning modeling framework

Machine learning analyses were conducted using R (version 4.5.1) within the tidymodels ecosystem. The study cohort (n = 240) was randomly partitioned into a training set and an independent test set at a 3:1 ratio. Stratified sampling based on the outcome variable was employed to ensure balanced class distribution across both subsets.

#### 2.7.2 Data preprocessing and feature engineering

Preprocessing was applied strictly to the training set to prevent data leakage. A unified recipe was constructed, involving: (1) exclusion of incomplete cases; (2) one-hot encoding for categorical predictors; and (3) Z-score standardization (centering and scaling) for numerical variables. For the Single-Layer Neural Network (SLNN), range normalization (min-max scaling) was applied instead of standardization.

#### 2.7.3 Model specification and hyperparameter tuning

Nine classification algorithms were implemented: Decision Tree (DT), Random Forest (RF), XGBoost, Regularized Logistic Regression (Lasso/Ridge/Elastic Net), Support Vector Machine with Radial Basis Function kernel (SVM-RBF), SLNN, LightGBM, K-Nearest Neighbors (KNN), and standard Logistic Regression (LR) as a baseline.

Hyperparameter tuning was applied to all models except the baseline LR using a grid search strategy with 10 candidate parameter sets. Optimization focused on maximizing the AUC. Internal validation employed 5-fold cross-validation repeated 5 times on the training set.

#### 2.7.4 Model training, evaluation, and validation strategy

Diagnostic models for CRC were developed using the training set. Model performance, including calibration and optimal decision thresholds, was initially evaluated via cross-validation. Final validation was conducted on the independent test set, which was processed using the training-derived preprocessing recipe. Generalization capability was assessed using Accuracy, Sensitivity, Specificity, Precision, F1-score, and AUC. The LR model served as a statistical baseline to quantify the performance gain of the machine learning algorithms.

## 3 Results

### 3.1 Prognostic Value and differential expression of GPX4 in CRC

**Fig. 2:**
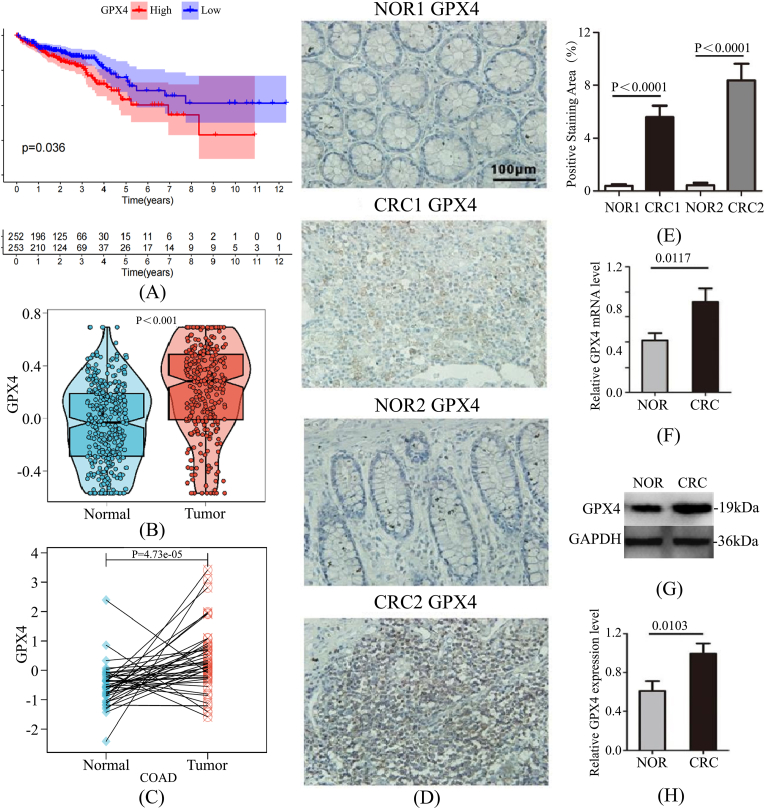
Comprehensive Analysis of GPX4 Expression and Prognostic Value in CRC. (A) Kaplan-Meier survival analysis comparing patients with high versus low *GPX4* expression.(B) Differential expression of *GPX4* in CRC versus normal tissues.(C) Pairwise comparison of *GPX4* expression in CRC and matched normal tissues.(D) Representative images showing differential GPX4 expression in tumor and adjacent tissues.(E) Statistical quantification of GPX4 immunohistochemical staining.(F) Relative GPX4 mRNA expression levels in CRC tissue samples.(G) Representative Western blotting bands of GPX4 protein in CRC tissues.(H) Quantitative analysis of relative GPX4 protein expression.

Survival analysis indicated that patients with low *GPX4* expression exhibited significantly better overall survival (OS) compared to those with high expression (Fig. 2A). Bioinformatic analysis of the TCGA dataset revealed that GPX4 levels were significantly elevated in CRC tissues relative to normal controls (Fig. 2B and C). Experimental validation using IHC, qRT-PCR, and Western blotting confirmed that GPX4 expression was markedly higher in tumor tissues than in paired adjacent normal tissues (Fig. 2D–H).

### 3.2 Single-cell expression of *GPX4* and Immune Cell Infiltration

**Fig. 3:**
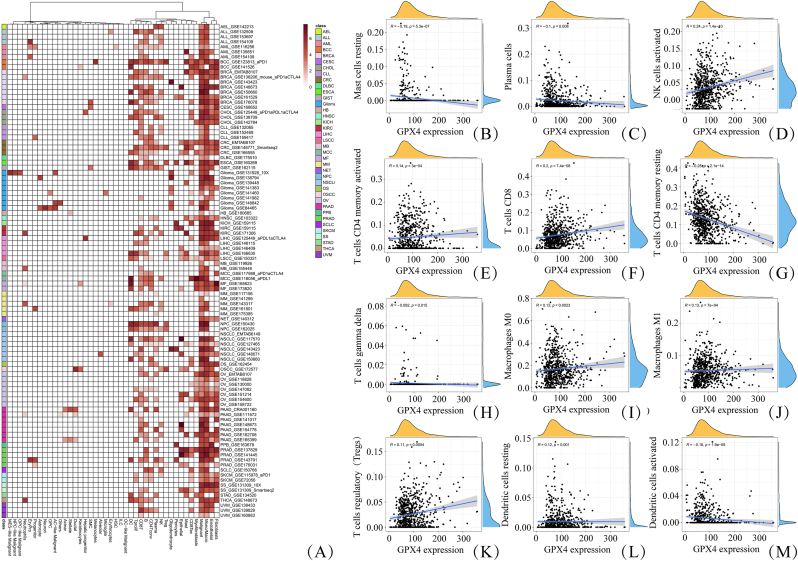
Single-Cell Analysis and Immune Infiltration Landscape of GPX4 in CRC. (A) Pan-cancer single-cell expression profile of GPX4. (B–M) Correlation analysis between GPX4 expression and the infiltration levels of 12 distinct immune cell types in CRC (Table 2).

Single-cell transcriptomic analysis (Fig. 3A) demonstrated that *GPX4* was highly expressed across several malignancies, including acute myeloid leukemia (AML), breast cancer, and CRC, while exhibiting greater heterogeneity in other cancer types. Within CRC, *GPX4* expression varied considerably across individual samples and datasets. At the cellular level, *GPX4* was detectable in multiple cell populations, with expression being notably enriched in macrophages compared to epithelial cells.

Immune infiltration analysis (Fig. 3B–M, Table 2) revealed significant correlations between *GPX4* expression and the abundance of 12 immune cell types. Specifically, *GPX4* expression was positively correlated with the infiltration of activated natural killer (NK) cells, CD8^+^ T cells, activated memory CD4^+^ T cells, M1 macrophages, M0 macrophages, regulatory T cells (Tregs), and resting dendritic cells. Conversely, negative correlations were observed with resting memory CD4^+^ T cells, resting mast cells, activated dendritic cells, plasma cells, and gamma-delta (γδ) T cells.

**Table 2:** Correlation between *GPX4* expression and immune cell infiltration in CRC.

Cell	R	P
NK cells activated	0.24	P < 0.001
T cells CD8	0.2	P < 0.001
T cells CD4 memory activated	0.14	P < 0.001
Macrophages M1	0.13	P < 0.001
Macrophages M0	0.12	0.0023
T cells regulatory (Tregs)	0.11	0.0054
T cells CD4 memory resting	0.28	P < 0.001
Mast cells resting	0.19	P < 0.001
Dendritic cells activated	0.16	P < 0.001
Plasma cells	0.1	0.008
Dendritic cells resting	0.12	0.001
T cells gamma delta	0.092	0.015

### 3.3 Assessment of normality and cohort characteristics

The Shapiro–Wilk test confirmed that all continuous variables analyzed in this study followed a normal distribution (Table 3). Baseline demographic and clinical characteristics of the cohort, including tumor stage and metastatic status, are summarized in Table 4.

**Table 3:** Results of normality tests for continuous variables.

Sample	Statistic	P value
Age - CRC Group	0.977	0.037
Age - Control Group	0.95	<0.001
Gender - CRC Group	0.554	<0.001
Gender - Control Group	0.799	<0.001
Serum iron - CRC Group	0.899	<0.001
Serum iron - Control Group	0.959	0.001
CA19-9 - CRC Group	0.238	<0.001
CA19-9 - Control Group	0.265	<0.001
CEA - CRC Group	0.297	<0.001
CEA - Control Group	0.375	<0.001
GPX4 - CRC Group	0.8	<0.001
GPX4 - Control Group	0.68	<0.001
Serum iron - male	0.928	<0.001
Serum iron - female	0.944	<0.001
CA19-9 - male	0.205	<0.001
CA19-9 - female	0.151	<0.001
CEA - male	0.248	<0.001
CEA - female	0.371	<0.001
GPX4 - male	0.773	<0.001
GPX4 - female	0.657	<0.001

**Table 4:** Demographic and clinical characteristics of the study population.

Sample	Groups	Number(n)	Percentage (%)
tumor stage	Ⅰ	30	30
	Ⅱ	17	17
	Ⅲ	44	44
	Ⅳ	10	10
metastatic status	0	69	69
	1	32	32

### 3.4 Diagnostic value of serum GPX4 in CRC patients

Baseline demographic characteristics were compared between the CRC group and the Control group (Table 5). While no significant difference was observed in gender distribution, patients in the CRC group were significantly older than those in the Control group.

**Table 5:** Comparison of demographic characteristics between the CRC and control groups.

Groups	Number of Cases	Age	Gender	
			male	female
CRC Group	120	63.22	74	46
Control Group	120	53.9	69	51
t/X^2^		7.265	0.433	
P		0.000	0.511	

### 3.5 Gender-specific analysis of serum markers

Comparison of serum markers revealed generally higher levels in male participants compared to females. Specifically, the differences in GPX4 and serum iron levels were statistically significant, whereas the elevations in CEA and CA19-9 did not reach statistical significance (Table 6).

**Table 6:** Comparison of serum CEA, CA19-9, GPX4, and iron levels stratified by gender.

Groups	Number of Cases	CEA (ng/mL)	CA19-9(U/mL)	GPX4 (pmol/mL)	Serum iron (μmol/L)
male	143	25.600	273.074	75.529	13.415
female	97	17.660	204.721	51.860	10.575
t		0.581	0.397	3.171	2.546
P		0.562	0.692	0.002	0.012

### 3.6 Comparison of serum markers between CRC and Control Groups

Serum levels of CEA, CA19-9, and GPX4 were significantly higher in the CRC group compared to the Control group. Conversely, serum iron levels were significantly lower in the CRC group (Table 7). All observed differences were statistically significant (P < 0.05).

**Table 7:** Comparison of serum CEA, CA19-9, GPX4, and iron levels between the CRC and control groups.

Groups	Number of Cases	CEA (ng/mL)	CA19-9(U/mL)	GPX4 (pmol/mL)	Serum iron (μmol/L)
CRC Group	120	42.170	436.781	92.157	10.391
Control Group	120	2.611	54.115	39.769	14.143
t		3.002	2.29	7.862	−3.464
P		0.003	0.023	<0.001	0.001

### 3.7 Multivariate analysis of diagnostic factors

Binary logistic regression was performed to evaluate the independent diagnostic value of serum markers while adjusting for age (Table 8). Age was identified as a significant independent risk factor for CRC. In the adjusted model, serum iron showed a significant inverse association with CRC, whereas CEA and GPX4 were independent positive predictors. Notably, CA19-9 did not retain statistical significance after adjusting for age. These results confirm that the diagnostic utility of GPX4, CEA, and serum iron is independent of age.

**Table 8:** Multivariate logistic regression analysis of serum markers and age for CRC prediction.

Variables in the Equation	B	Standard Error	Wald	Degrees of Freedom	Significance	Exp(B)	95% Confidence Interval for Exp(B)	
							Lower limit	Upper limit
Serum iron	−0.091	0.025	13.587	1	<0.001	0.913	0.870	0.958
CA19-9	0.001	0.001	2.731	1	0.098	1.001	1	1.002
CEA	0.082	0.038	4.578	1	0.032	1.086	1.007	1.170
GPX4	0.029	0.006	25.073	1	<0.001	1.030	1.018	1.042
Age	0.081	0.020	15.844	1	<0.001	1.084	1.042	1.129

### 3.8 The diagnostic value of CEA, CA19-9, GPX4 and serum iron in CRC was analyzed

**Fig. 4:**
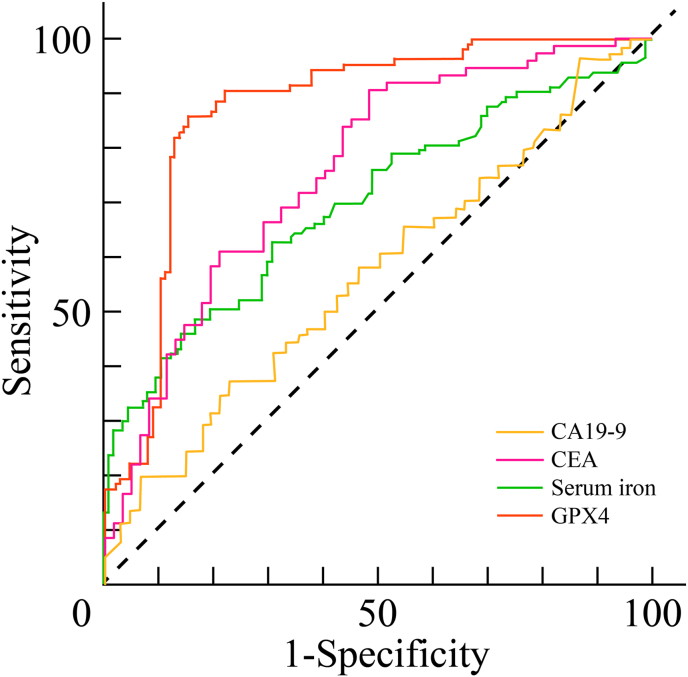
Roc curves evaluating the diagnostic performance of serum markers.

The diagnostic efficacy of CEA, CA19-9, GPX4, and serum iron was evaluated using ROC curve analysis (Fig. 4). The symmetric discriminative index (P_sym_) was calculated to quantify discriminative ability independent of class labels. Results demonstrated that all four markers possessed significant diagnostic value for CRC (Table 9).

**Table 9:** ROC analysis and diagnostic metrics of individual serum markers in CRC.

Test Variable	Area Under Curve (AUC)	P_sym_	Sensitivity (%)	Specificity (%)	Standard Error	Cut-off Value	Asymptotic Significance	95% Confidence Interval	
								Lower limit	Upper limit
Serum iron	0.361	0.639	0.581	0.567	0.036	7.585	0	0.291	0.431
CA19-9	0.576	0.576	0.562	0.583	0.037	14.32	0.042	0.504	0.648
CEA	0.722	0.722	0.707	0.767	0.033	2.335	0	0.658	0.786
GPX4	0.873	0.873	0.684	0.812	0.024	42.921	0	0.826	0.92

### 3.9 Diagnostic efficacy of combined testing strategies

Optimal cutoff values were determined via ROC analysis: serum iron <7.59 μmol/L, CA19-9 > 14.32 U/mL, CEA >2.34 ng/mL, and GPX4 > 42.92 pmol/ml. Based on these thresholds, the sensitivity and specificity of individual markers were established. Furthermore, serial and parallel diagnostic strategies were evaluated, with agreement assessed using the Kappa test (Table 10).

**Table 10:** Diagnostic performance of serial and parallel combined testing strategies in CRC.

Diagnosis Methods	sensitivity (%)	specificity (%)	Accuracy Rate	Kappa coefficient
Serum iron in combination with CEA	0.45	0.942	0.696	0.258
GPX4 in combination with CEA	0.675	0.958	0.817	0.550
Serum iron in combination with GPX4 and CEA	0.833	0.75	0.792	0.492
Serum iron in combination with GPX4 and CA19-9	0.708	0.833	0.771	0.417

### 3.10 Development and validation of machine learning diagnostic models

#### 3.10.1 Preliminary validation

**Fig. 5:**
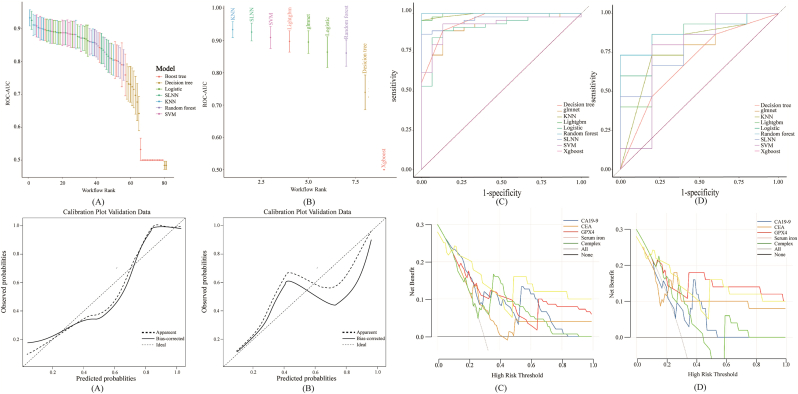
Evaluation of Model Performance in the Preliminary Experiment.(A) Comparison of AUC values for all models in the preliminary phase.(B) AUC of the best-performing model.(C) ROC curves for the training set.(D) ROC curves for the testing set.(E) Calibration curves for the training set.(F) Calibration curves for the testing set.(G) Decision Curve Analysis (DCA) for the training set.(H) Decision Curve Analysis (DCA) for the testing set.

Prior to formal model development, stratified sampling was applied to generate a subset for preliminary validation. The results indicated that the models demonstrated promising performance and calibration, justifying further comprehensive analysis (Fig. 5).

#### 3.10.2 Model selection and construction

**Fig. 6:**
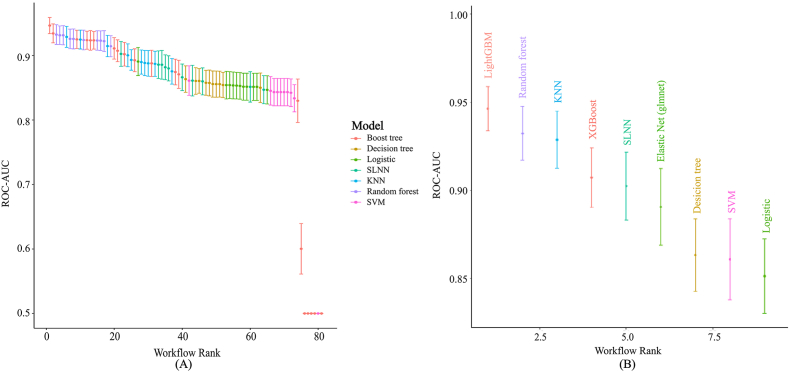
Comparative Analysis of Machine Learning Models.(A) Comparison of diagnostic performance across all nine algorithms.(B) Quantification of the AUC value for the optimal model.

In the formal experimental phase, nine machine learning algorithms were evaluated using cross-validation. The diagnostic performance of these models was systematically compared (Fig. 6A), and the AUC value of the optimal model was quantified (Fig. 6B).

Following model selection, the entire cohort (n = 240) was randomly partitioned into a training set (n = 180) and an independent test set (n = 60) at a 3:1 ratio.

#### 3.10.3 Performance in the training set

**Fig. 7:**
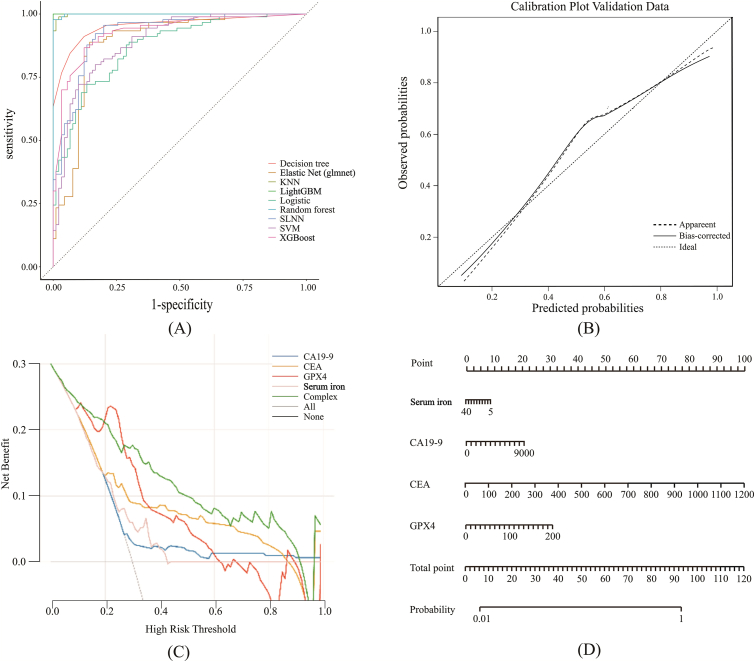
Performance Evaluation of the Optimal Diagnostic Model in the Training Set. (A) ROC curves of the model in the training cohort.(B) Calibration plot assessing the agreement between predicted probabilities and observed outcomes in the training set.(C) Decision Curve Analysis (DCA) estimating the clinical net benefit in the training set.(D) Nomogram constructed for CRC risk prediction based on the training data.

The optimal diagnostic model was developed using the training set (Table 11). The ROC curves demonstrated robust discriminatory ability (Fig. 7A). Calibration plots indicated good agreement between predicted probabilities and observed outcomes (Fig. 7B). Decision Curve Analysis (DCA) highlighted the clinical net benefit of the model (Fig. 7C), and a nomogram was constructed to facilitate clinical application (Fig. 7D).

**Table 11:** Evaluation results of the model in the training and validation cohort.

Model	Dataset	Accuracy (%)	Sensitivity (%)	Specificity (%)	F1-score	AUC
Logistic	Training Set	80.6	81.1	80	0.807	0.871
	Test Set	83.3	80	86.7	0.828	0.886
Random forest	Training Set	98.9	97.8	100	0.989	0.999
	Test Set	90	83.3	96.7	0.893	0.909
Single-Layer Neural Network (SLNN)	Training Set	87.8	93.3	82.2	0.884	0.924
	Test Set	86.7	80	93.3	0.857	0.889
Support Vector Machine (SVM)	Training Set	81.7	80	83.3	0.814	0.893
	Test Set	88.3	76.7	100	0.868	0.882
XGBoost	Training Set	87.2	86.7	87.8	0.872	0.929
	Test Set	83.3	83.3	83.3	0.833	0.873
Decision tree	Training Set	89.4	91.1	87.8	0.896	0.954
	Test Set	80	83.3	76.7	0.806	0.847
Elastic Net (glmnet)	Training Set	87.2	88.9	85.6	0.874	0.889
	Test Set	81.7	80	83.3	0.814	0.833
K-Nearest Neighbors (KNN)	Training Set	98.3	98.9	97.8	0.983	0.999
	Test Set	88.3	86.7	90	0.881	0.86
LightGBM	Training Set	100	100	100	1	1
	Test Set	90	83.3	96.7	0.893	0.911

#### 3.10.4 Validation in the independent test set

**Fig. 8:**
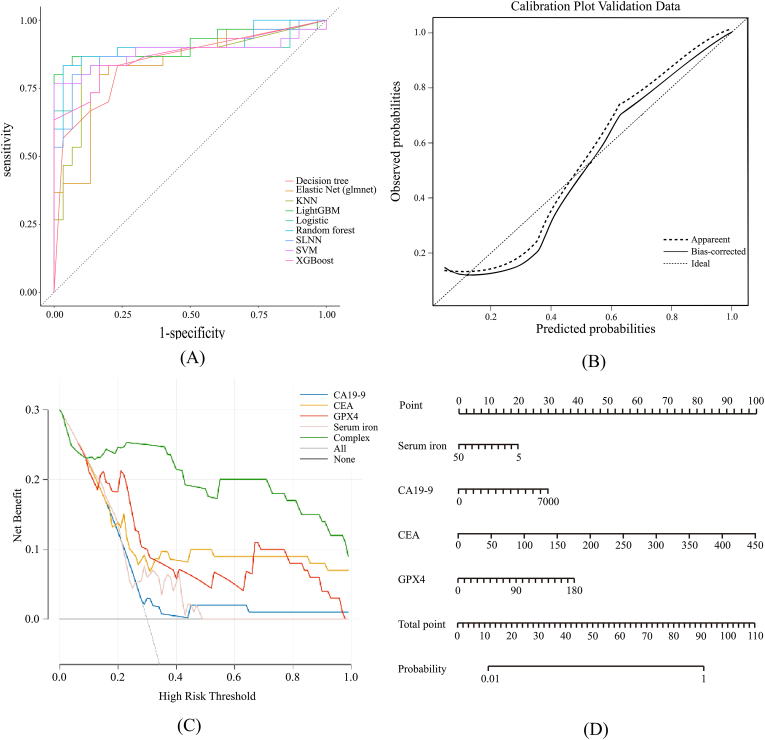
Validation of the Optimal Diagnostic Model in the Independent Test Set.(A) ROC curves of the model in the independent testing cohort.(B) Calibration plot assessing the model's accuracy in the test set.(C) Decision Curve Analysis (DCA) confirming clinical utility in the test set.(D) Evaluation of the nomogram's predictive performance in the test set.

To assess generalization capability, the model was validated on the independent test set (n = 60). Consistent with the training results, the test set exhibited high AUC values (Fig. 8A) and satisfactory calibration (Fig. 8B). DCA confirmed the clinical utility of the model in the validation cohort (Fig. 8C), further supporting the predictive accuracy of the nomogram (Fig. 8D).

## 4 Discussion

Ferroptosis, a regulated cell death process characterized by iron-dependent lipid peroxidation, has emerged as a critical mechanism in tumor suppression. As the central regulator of this process, GPX4 plays a pivotal role in modulating tumor initiation, progression, and therapeutic response. Accumulating evidence suggests that GPX4 acts as an oncogenic driver in various malignancies. For instance, GPX4 inactivation has been shown to sensitize radioresistant cancer cells to ionizing radiation [26] and inhibit migration in renal clear cell carcinoma [27]. Similarly, pharmacological inhibition of GPX4 suppresses glioma growth via ferroptosis induction [28].

Consistent with these observations, our multi-dimensional analysis—integrating bioinformatics with experimental validation via IHC, qRT-PCR, and Western blotting—demonstrated that GPX4 is significantly upregulated in CRC tissues compared to matched adjacent normal tissues. This upregulation aligns with expression patterns documented in other malignancies [22,[29], [30], [31]]. Crucially, our survival analysis revealed a significant inverse correlation between GPX4 expression and overall survival, identifying high GPX4 levels as a predictor of poor prognosis in CRC patients.

Metabolically, the rapid proliferation of cancer cells generates elevated levels of reactive oxygen species (ROS) and lipid peroxides. To mitigate this oxidative stress, tumor cells often overexpress GPX4, which reduces lipid hydroperoxides to non-toxic alcohols, thereby preventing ROS-mediated ferroptotic cell death and promoting survival [31].

Our single-cell and immune infiltration analyses revealed significant heterogeneity in GPX4 expression within the CRC microenvironment. Notably, elevated GPX4 levels were associated with increased infiltration of specific immune effector cells, including natural killer (NK) cells, M0 macrophages, and CD8^+^T cells. This positive correlation suggests that while immune cells infiltrate the tumor, high GPX4 expression may serve as an adaptive mechanism, enabling tumor cells to resist immune-mediated ferroptosis. Immunohistochemical validation confirmed that GPX4 is predominantly localized within tumor parenchymal cells. Collectively, these findings suggest that GPX4 not only facilitates CRC progression but also modulates the local immune landscape, highlighting its potential as both a diagnostic biomarker and a therapeutic target.

CRC remains a leading cause of cancer-related mortality worldwide [32]. Early identification and accurate diagnosis are paramount for improving patient prognosis. Current diagnostic modalities, including abdominal ultrasound, computed tomography (CT), positron emission tomography-CT (PET-CT), magnetic resonance imaging (MRI), and serological testing, form the cornerstone of clinical assessment. However, these methods often lack sufficient sensitivity for early-stage detection, leading to a substantial proportion of patients being diagnosed at advanced stages. Consequently, there is an urgent need to develop novel, high-precision diagnostic models for CRC.

CEA, an acidic oncofetal glycoprotein, is a well-established tumor marker widely utilized in the diagnosis, monitoring, and prognostic assessment of CRC [33,34]. While valuable, its diagnostic accuracy can be influenced by baseline levels [35], and its utility often extends to predicting recurrence patterns in gastrointestinal malignancies [36].

Similarly, CA19-9, a biomarker specific to glandular epithelia, is frequently overexpressed in pancreatic [[37], [38], [39]]、gastric cancer [40,41]、CRC [[42], [43], [44]]and other adenocarcinomas. As a marker specific for glandular epithelium, CA19-9 is frequently overexpressed in gastrointestinal tumors [44] and has become a key clinical indicator for several malignancies. Despite their widespread clinical application, both CEA and CA19-9 are recognized to have only moderate sensitivity and specificity, particularly for early-stage detection [45,46].

To overcome these limitations, we integrated the ferroptosis-related markers GPX4 and serum iron with these conventional markers. Our evaluation using traditional statistical methods demonstrated that the combination of GPX4 and CEA achieved a notably high diagnostic accuracy. This finding highlights the potential of integrating metabolic and ferroptosis-related biomarkers with traditional antigens to establish a more robust strategy for the early diagnosis of CRC.

To capitalize on the diagnostic potential of integrating serum ferroptosis markers with conventional indices, we employed multiple machine learning algorithms to construct and evaluate a high-precision diagnostic model. AUC was utilized as the primary metric for performance assessment. Among the evaluated algorithms, XGBoost, Random Forest, and SVM demonstrated superior performance during cross-validation, achieving AUC values exceeding 0.85 (Fig. 6B). In the training cohort, the ROC curves for these models indicated excellent discriminatory ability, effectively balancing sensitivity and specificity. Conversely, Decision Tree, KNN, and Logistic Regression models yielded comparatively lower predictive accuracy. Crucially, the optimal model maintained high diagnostic accuracy in the independent test set (Fig. 8), confirming its robustness and generalizability for CRC detection.

Calibration analysis was conducted to evaluate the agreement between predicted probabilities and observed outcomes. The training set exhibited satisfactory calibration, aligning well with the ideal diagonal. However, minor deviations were observed in the test set. While the formal model demonstrated superior calibration compared to the preliminary validation model, these discrepancies may be attributed to the limited sample sizes of both cohorts. Consequently, future multi-center studies with larger sample sizes are warranted to further refine model calibration and validate these findings.

DCA was performed to evaluate the clinical utility of the predictive models by quantifying the net benefit across a range of threshold probabilities. In the training cohort (Fig. 7C), GPX4 demonstrated the highest net benefit at lower threshold probabilities, highlighting its potential for early-risk stratification. This pattern was consistently observed in the validation cohort (Fig. 8C). However, at higher risk thresholds, the net benefit of GPX4 diverged from that of CEA. We attribute this variation to the limited sample size and the inherent heterogeneity of GPX4 expression levels between healthy individuals and CRC patients.

Synthesizing the findings from AUC analysis, calibration assessment, and DCA, we conclude that despite minor deviations in calibration, the proposed machine learning model demonstrates robust discriminative ability and significant clinical utility for CRC diagnosis.

The clinical trade-off between the performance gains of machine learning and the inherent transparency of logistic regression warrants careful evaluation. Logistic regression offers interpretable risk quantification through variable weights, a fundamental advantage for clinical decision-making. While ensemble models can provide post-hoc explanations via methods like SHAP, the stability and clinical accountability of these interpretations remain practical challenges. In this study, the primary value of machine learning lies not in immediately replacing interpretable linear models, but in identifying complex variable interactions that conventional methods often overlook. These insights provide a roadmap for future research, which should aim to incorporate these interaction effects into a transparent logistic regression framework, thereby balancing diagnostic performance with clinical interpretability.

This study demonstrates originality across conceptual and methodological dimensions. First, we provide the first systematic assessment of serum GPX4—a central regulator of ferroptosis—as a diagnostic biomarker for CRC, confirming its robust performance as an independent indicator. Methodologically, we moved beyond single-marker approaches by innovatively integrating GPX4 and serum iron with the conventional markers CEA and CA19-9. By refining this multi-parameter panel with machine learning algorithms, we constructed a diagnostic model with significantly enhanced performance. This strategy of "multi-marker integration coupled with intelligent modeling" establishes a novel methodological framework for the non-invasive, early detection of CRC.

Furthermore, our study is the first to reveal a significant correlation between GPX4 expression and the infiltration of specific immune cell subsets in CRC. This finding suggests that GPX4 may function as a molecular bridge linking tumor cell ferroptosis to the tumor immune microenvironment. This insight offers a novel perspective on CRC progression and highlights GPX4 as a potential target for immunotherapeutic interventions.

These findings hold substantial translational promise. Clinically, our multi-marker diagnostic model serves as an effective non-invasive adjunct to colonoscopy. It is particularly valuable for large-scale population screening, especially in resource-constrained settings, where it could significantly improve early diagnosis rates. Regarding disease management, longitudinal monitoring of these markers may facilitate the assessment of treatment response and recurrence risk, potentially serving as a companion diagnostic for emerging ferroptosis-inducing therapies.

From a broader perspective, the multidimensional integration framework established herein paves the way for incorporating future multi-omics data, advancing CRC management toward precision medicine. Ultimately, the widespread adoption of this accurate, accessible, and cost-effective serological strategy could reduce the societal burden of CRC, yielding significant public health benefits.

Several limitations of this study warrant acknowledgement. First, despite adjusting for age in the logistic regression analysis to confirm the independent diagnostic value of GPX4, CEA, and serum iron, the retrospective case-control design inherently precludes the complete elimination of confounding. Residual confounding from unmeasured variables—such as lifestyle factors, dietary habits, genetic background, and systemic inflammatory status—cannot be ruled out.

Second, given that multiple statistical comparisons were performed without strict adjustment for Type I error, our findings should be interpreted as exploratory and require confirmation in independent cohorts.

Third, data were derived from a single center, a constraint largely necessitated by the technical standardization required for GPX4 measurement. Consequently, the generalizability and robustness of the diagnostic model to other populations or healthcare settings remain to be established through external validation.

Future research should prioritize prospective, multicenter studies with larger, age-matched cohorts to validate these findings and ensure reliability across diverse clinical contexts. Furthermore, exploring the integration of additional emerging biomarkers may offer opportunities to further enhance diagnostic precision.

## 5 Conclusion

This study elucidates the critical role of ferroptosis in the pathogenesis and progression of CRC. We confirmed that GPX4, a central regulator of ferroptosis, is significantly upregulated in CRC tissues and serves as a robust predictor of poor prognosis. Furthermore, multi-omics analysis revealed a significant correlation between GPX4 expression and immune infiltration, suggesting its potential involvement in modulating the tumor immune microenvironment.

Diagnostically, the machine learning model constructed by integrating GPX4 and serum iron with traditional markers demonstrated superior performance in both training and independent validation cohorts. This combined model significantly outperformed individual biomarkers, highlighting its potential for clinical translation.

Collectively, this study presents a novel, non-invasive diagnostic strategy. By combining ferroptosis-related markers with intelligent computational modeling, we provide a clinically significant tool for the early detection and risk stratification of CRC.

## Ethics approval and consent to participate

This study was approved by the Ethics Committee of the First Affiliated Hospital of Jinzhou Medical University (Approval No. 202127). Informed consent was obtained from all participants in accordance with the Declaration of Helsinki,and all experiments were conducted in accordance with relevant guidelines and regulations.

## Consent for publication

Written informed consent for participants was not required for this study in accordance with national legislation and institutional requirements.

## Funding

This work was supported by the Science and Technology Research Joint Project of Liaoning Provincial Department, 2023-MSLH-055; The funding of Scientific Research of The First Affiliated Hospital of Jinzhou Medical University KYTD-2022006. The Undergraduate Innovation and Entrepreneurship Training Program Project of Liaoning Province, S202310160031.

## CRediT authorship contribution statement

**Chao Mei:** Conceptualization, Data curation, Formal analysis, Methodology, Software, Writing – original draft, Writing – review & editing. **Jianfeng Shi:** Investigation, Resources, Supervision. **Hanxin Liu:** Formal analysis. **Xiao Liu:** Investigation. **Xiang Feng:** Validation. **Chenbo Chen:** Investigation. **Zixin Wang:** Supervision. **Wenjie Pan:** Data curation. **Shuo Bai:** Conceptualization, Validation. **Li Zhang:** Funding acquisition. **Yang Li:** Funding acquisition.

## Declaration of competing interest

The authors declare that they have no known competing financial interests or personal relationships that could have appeared to influence the work reported in this paper.

## Data Availability

Data will be made available on request.
