# Supplementary material for: Enhancing colorectal cancer diagnosis with the ferroptosis marker GPX4 and serum biomarkers: A retrospective analysis and machine learning approach
Source: Biochem Biophys Rep. 2026 Feb 24;45:102517. doi: 10.1016/j.bbrep.2026.102517 (PMC12955147; doi:10.1016/j.bbrep.2026.102517)

| \| Forward primer Sequence (5'->3') \| Reverse primer Sequence (5'->3') \| \| --- \| --- \| \| TCACCAAGTTTGGACACCGT \| ATAGTGGGGCAGGTCCTTCT \|   GPX4 Primers |
| --- | --- | --- | --- | --- |

**Input PCR template** [NM_001039847.3](https://www.ncbi.nlm.nih.gov/entrez/viewer.fcgi?db=nucleotide&id=1540583208) Homo sapiens glutathione peroxidase 4 (GPX4), transcript variant 2, mRNA

**Range** 1 - 873

**Specificity of primers** Primer pairs are specific to input template as no other targets were found in selected database: Refseq mRNA (Organism limited to Homo sapiens)

product length = 117

Forward primer 1 TCACCAAGTTTGGACACCGT 20

Template 544 .................... 563

Reverse primer 1 ATAGTGGGGCAGGTCCTTCT 20

Template 660 .................... 641


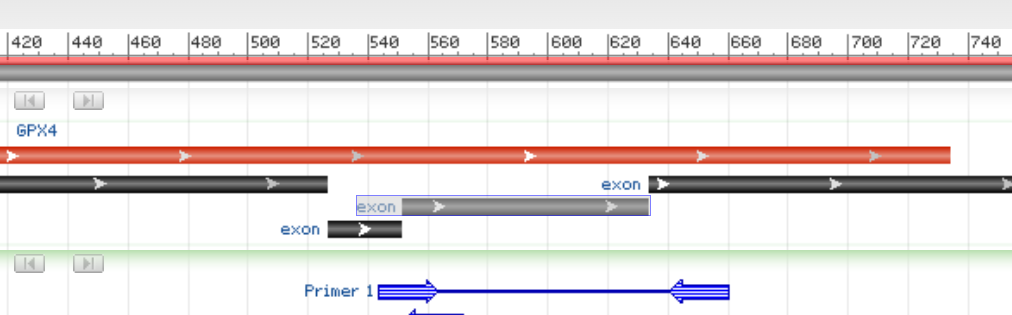


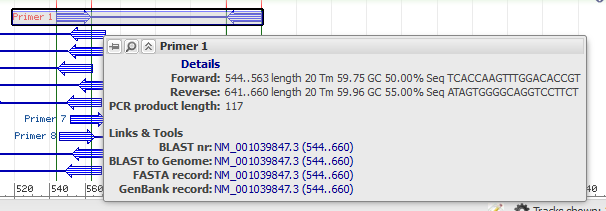


GPX4 Western Blotting


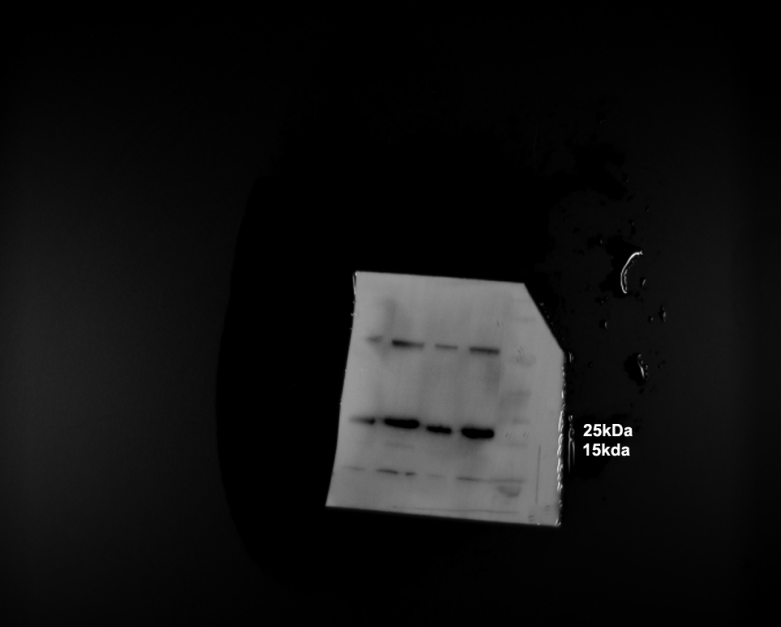


GAPDH Western Blotting


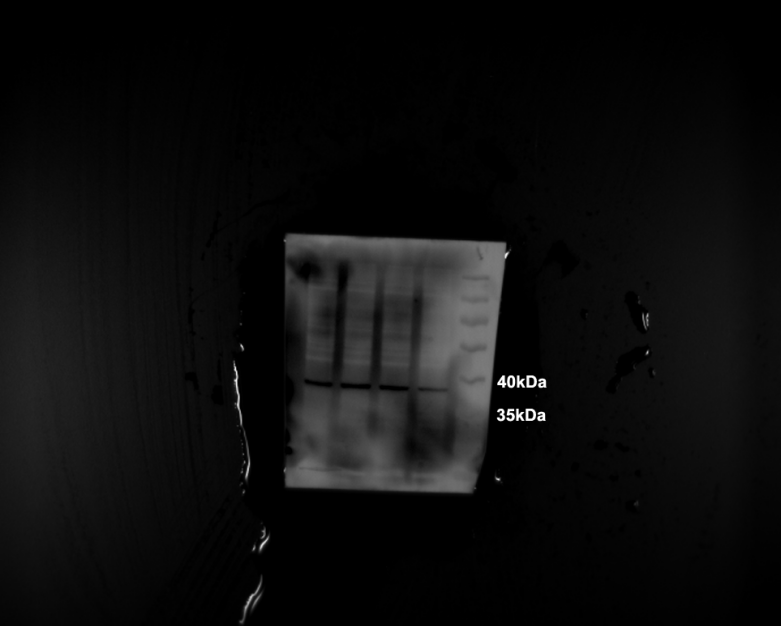

Supplement: Multimedia component 1 [file mmc1.docx]
